# BBH-LS: an algorithm for computing positional homologs using sequence and gene context similarity

**DOI:** 10.1186/1752-0509-6-S1-S22

**Published:** 2012-07-16

**Authors:** Melvin Zhang, Hon Wai Leong

**Affiliations:** 1 School of Computing, National University of Singapore, Computing 1, 13 Computing Drive, Singapore 117417, Republic of Singapore

## Abstract

**Background:**

Identifying corresponding genes (orthologs) in different species is an important step in genome-wide comparative analysis. In particular, one-to-one correspondences between genes in different species greatly simplify certain problems such as transfer of function annotation and genome rearrangement studies. Positional homologs are the direct descendants of a single ancestral gene in the most recent common ancestor and by definition form one-to-one correspondence.

**Results:**

In this work, we present a simple yet effective method (BBH-LS) for the identification of positional homologs from the comparative analysis of two genomes. Our BBH-LS method integrates sequence similarity and gene context similarity in order to get more accurate ortholog assignments. Specifically, BBH-LS applies the bidirectional best hit heuristic to a combination of sequence similarity and gene context similarity scores.

**Conclusion:**

We applied our method to the human, mouse, and rat genomes and found that BBH-LS produced the best results when using both sequence and gene context information equally. Compared to the state-of-the-art algorithms, such as MSOAR2, BBH-LS is able to identify more positional homologs with fewer false positives.

## Background

Genome-wide comparative analysis of different species is only possible if we can identify conserved elements across species boundaries [1]. For many studies, the elements under consideration are the set of protein coding genes. Therefore, the identification of corresponding genes between different species is an important step in any genome-wide comparative analysis. In particular, one-to-one correspondences between genes in different species are preferred in certain applications such as transfer of function annotation [2] and genome rearrangement studies [3] as they greatly simplify subsequent analysis.

**Figure 1:**
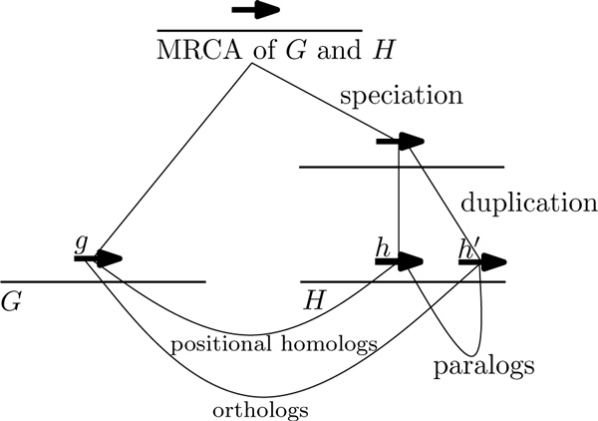
**Gene tree for three gene showing the different types of homologs**. The gene tree for three genes *g*, *h*, and *h*' that descended from a single ancestral gene in the most recent common ancestor (MRCA) of genome *G *and *H*. Gene *g *is orthologous to both *h *and *h*', but only *g *and *h *are positional homologs because *h *is the direct descendant of the ancestral gene. Genes *h *and *h*' are paralogs as they are separated by a duplication event.

Consider a set of extant genomes and their most recent common ancestor (MRCA). For each gene in the MRCA, there is at most one direct descendant of the gene in each of the extant genomes. The direct descendants of a gene in the MRCA form a set of *positional homologs *[4]. A single ancestral gene may have multiple descendants due to gene duplication, or no descendants because of gene loss. In the case of gene duplication, we distinguish between the gene that remains in the original location and the copy inserted into a new location. The gene that retains its ancestral location is the direct descendant. Positional homologs represent a set of genes in one-to-one correspondence with each other where each member best reflect the original location of the ancestral gene in the MRCA. Similar concepts in the literature include exemplars [3], ancestral homologs [5], and main orthologs [6]. Orthologs are genes separated by a speciation event, while paralogs are genes separated by a duplication event. Orthologs and paralogs together make up the set of homologs [7]. Positional homologs are a subset of orthologs. Figure 1 shows the gene tree for three genes found in two genomes and it illustrates the concept of positional homologs, orthologs, and paralogs.

The problem of finding the set of positional homologs between two genomes is known as the ORTHOLOG ASSIGNMENT problem [6]. Current methods for the ORTHOLOG ASSIGNMENT problem fall into three categories: distance minimization, similarity maximization, and rule-based. *Distance minimization *methods relies on the parsimony principle. They assume that the removal of all the genes except for the positional homologs minimizes the genomic distance (usually some form of edit distance with genomic operations) between two genomes. Genomic distance measures such as the reversal distance [8] and breakpoint distance [9] have been considered using a branch-and-bound approach [3] as the corresponding computational problems are NP-hard [10]. MSOAR2 [11] uses a number of heuristic algorithms to assign positional homolog pairs in several phases to minimize the number of reversals, translocations, fusions, fissions, and gene duplications between two genomes.

Closely related to distance minimization are the *similarity maximization *approaches. By identifying conserved structures between genomes, we can determine the similarity between them. We can model the ORTHOLOG ASSIGNMENT problem as finding the set of positional homologs that maximize the degree of similarity between two genomes. Bourque *et al. *[5] uses heuristics for the MAX-SAT problem to maximize the number of common or conserved intervals. The problem of maximizing the number conserved intervals is NP-hard [12]. Blin *et al. *[13] proposed a greedy method based on algorithms for global alignment that first finds a set of anchors and then recursively match genes found in large common intervals.

All of the preceding methods need a pre-processing step to compute gene families. This is typically accomplished using sequence similarity search followed by clustering of similar genes [14]. After that, sequence similarity is essentially reduced to a simple binary relation; two genes are the equivalent if they are in the same gene family and different otherwise. The main step uses heuristics to find a subset of genes that optimizes an NP-hard problem on gene orders. In short, the preceding methods use sequence similarity to build gene families and gene order information to further refine the gene families to get one-to-one gene matchings.

In contrast, *rule-based *methods do not need to build gene families. A widely used method for finding pairwise orthologs based on sequence similarity is the *bidirectional best hit (BBH) *heuristic. Two genes *g *and *h *form bidirectional best hits if the similarity between *g *and *h *is greater than that between *g *and any other gene (*h *is the best hit for *g*) and vice versa. In [4], a pair of BBHs are positional homologs if they are next to another pair of BBHs. Subsequently, [15] relaxed this condition and defined a local synteny test to determine whether a given pair of genes is a positional homolog pair. A gene pair passes the local synteny check if there are at least two pairs of genes (excluding the gene pair being tested) nearby with a sequence similarity above a certain threshold. Note that the local synteny test does not consider the sequence similarity between the gene pair being tested. Since positional homologs are a subset of all orthologs, other rule based methods designed for finding orthologs [16,17] can also be used to identify positional homologs by restricting ourselves to one-to-one orthologous groups.

So far, existing methods have used sequence similarity information and gene order separately. In this paper, we propose to combine sequence similarity and gene context similarity into a single similarity score and identify positional homologs using the bidirectional best hit heuristic.

This has the advantage that the method is easy to implement and computationally efficient. Furthermore, we can easily vary the weightage of each type of similarity. We expected sequence similarity to play a larger role. Surprisingly, we get the best results using equal weightage for sequence and gene context similarity. Our method outperforms more complex methods, such as MSOAR2, in identifying positional homologs between human, mouse, and rat.

## Methods

Our approach is to approximate positional homologs as bidirectional best hits using a scoring scheme that integrates both sequence and gene context similarity scores.

Bidirectional (or reciprocal) best hits (BBH) is a widely used heuristic for finding orthologs between two species. [18] compared a number of ortholog inference algorithms and found that BBH's overall performance is surprisingly good despite the simplicity of the method. In particular, they found that orthologs predicted by BBH show close functional relatedness. Another advantage of BBH is that it is easy to compute and commonly used in the literature.

**Figure 2:**
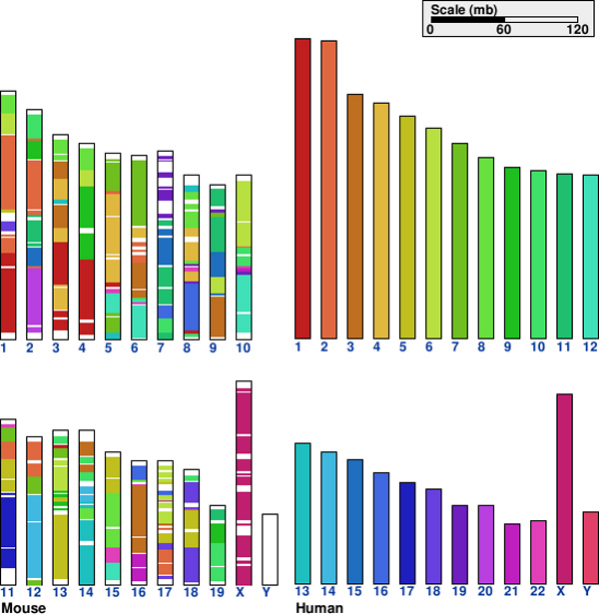
**Clustering of homologous genes between human and mouse**. Clusters of homologous genes form conserved synteny blocks between human and mouse genome. Generated by the Cinteny web server [26].

However, using sequence similarity alone is *not enough *to identify the positional homolog among several orthologs [4]. In such cases, gene context can be used to disambiguate between the paralogs because positional homologs tend to have more similar gene context as evidenced by the presence of large synteny blocks (see Figure 2).

Furthermore, [19] showed that in 29-38 percent of the orthologs they investigated in bacteria, the gene pair with the lower sequence similarity have a higher gene context similarity. Hence, they advised combining gene context information with protein sequence information to predict functional orthologs. In this work, we integrate sequence similarity score with a gene context similarity score that reflects the shared gene neighborhood between two genes.

In the following subsections, we give the details for computing sequence and gene context similarity scores and explain how to combine them to compute bidirectional best hits.

### Computing sequence similarity scores

We define the sequence similarity score between two genes as the Smith-Waterman alignment score between the respective peptide sequences. As a gene may have multiple transcripts, we use the transcript with the longest peptide sequence to represent the gene. We use the SSEARCH program from the FASTA v36 package [20] to compute the Smith-Waterman alignment score between all pairs of peptide sequences using default parameters optimized for high sensitivity (BLOSUM50 substitution matrix and E-value cutoff of 10).

We use peptide sequences as the basis of sequence comparison as they have a number of advantages over using nucleotide sequences [21]. Peptide sequences are not affected by synonymous substitution and hence able to detect more distant homology. Furthermore, the alignments are faster to compute since the peptide sequence is only one third the length of the nucleotide sequence. Heuristic search algorithms, such as BLAST, are often used to find homologous sequences since they avoid computing the expensive dynamic programming alignment. However, a serious drawback is that the derived scores (bit score or E-value) are not symmetric and are difficult to easily integrate with other scores. On the other hand, the Smith-Waterman alignment score is symmetric and modern implementations are sufficiently fast for our purpose.

Since we want to integrate both sequence similarity and gene context similarity scores, we normalize the Smith-Waterman scores so that it ranges from 0 to 1 with a score of 1 indicating maximum sequence similarity. The Smith-Waterman alignment score is roughly linearly proportional to the length of the peptide sequences compared; longer peptide sequences tend to have higher alignment scores. Therefore, we remove this dependence on the length of the peptide sequence and normalize the score to range between 0 and 1 by dividing by the maximum Smith-Waterman score of the two self alignments. We formally defined the normalized Smith-Waterman score, sw_norm_, as follows:

$$\text{s}{\text{w}}_{\text{norm}}\left(g,h\right)=\frac{\text{sw}\left(g,h\right)}{\text{max}\left\{\text{sw}\left(g,g\right),\text{sw}\left(h,h\right)\right\}}$$

where sw(*g*, *h*) is the Smith-Waterman alignment score between the peptide sequences of genes *g *and *h*.

### Computing gene context similarity scores

Gene context similarity refers to the similarity in the genomic context of two genes. In contrast to sequence similarity, there is no widely accepted method to determine the level of gene context similarity between two genes. In this work, we make use of the concept of *local synteny *proposed in [15,19].

Jun *et al. *[15] proposed a *local synteny test *that considers three genes upstream and downstream of two genes of interest to decide if they are orthologs. They modelled the sequence similarity between the two sets of six genes as a bipartite graph; there is an edge between two genes if their BLASTP E-value is less than 1*e*^-^^5^. They then compute a maximum matching of the graph. Two genes are *putative orthologs *if the size of the maximum matching is greater than one. In other words, they test if there is at least two other matching gene pairs in the vicinity of the gene pair of interest. They determine the BLASTP threshold and size of gene neighborhood by finding the values that maximizes the agreement with InParanoid [17] and Ensembl Compara [22] orthologs. They found that 93 percent of sampled inter-species pairs in five mammalian genomes (human, chimpanzee, mouse, rat, and dog) identified by their local synteny test are also found by InParanoid. By analyzing the remaining seven percent of the pairs, they conclude that the use of a local synteny test can resolve ambiguous many-to-many orthologous groups into one-to-one pairs. While the binary test proposed in [15] detects the presence of other matching gene pairs in the vicinity of *g *and *h*, it does not capture the *strength *of the gene context similarity nor does it make use of the sequence similarity of the gene pair being tested. Thus, it may cause errors in special cases: (a) *false positives *when the local context similarity is high, but the sequence similarity is low, or (b) *false negatives *when the local context similarity is low (only one other matching pair), while the sequence similarity is high.

**Figure 3:**
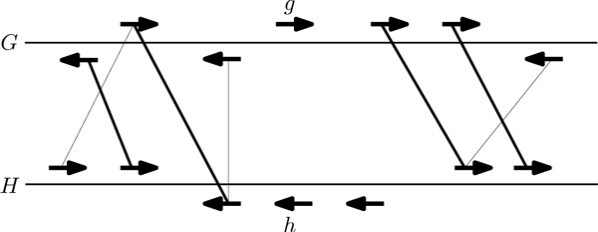
**Computing the local synteny score for *g *and *h***. We consider three genes upstream and downstream of the two genes of interest and add an edge between two genes if their BLASTP E-value is less than 1*e*^-5^. The thick edges show one of the possible maximum matching. The local synteny score of *g *and *h *is 4 since there are 4 edges in the maximum matching.

We define the *local synteny score*, lss(*g*, *h*), as an extension of the binary test proposed in [15], to capture the degree of gene context similarity between *g *and *h*. The *local synteny score *of two genes *g *and *h *is the size of the maximum matching between the six genes surrounding *g *and *h *(see Figure 3). This gives us a number between 0 and 6, which we normalize by dividing by 6. This is similar to the gene-neighborhood conservation score [19].

Formally, we define the normalized local synteny score, lss_norm_, as follows:

$$\text{ls}{\text{s}}_{\text{norm}}\left(g,h\right)=\frac{\left|\text{max}\text{matching}\;\text{of}\;\text{graph}\;G=\left(U\cup V,E\right)\right|}{6}$$

where *U *is the set of six genes around *g*, *V *is the set of six genes around *h *and there is an edge (*u*, *v*) in *E *if the BLASTP E-value of *u *and *v *is less than 1*e*^-5^.

### Combining sequence and gene context similarity and computing bidirectional best hits

Given the normalized sequence similarity scores (sw_norm_) and normalized gene context similarity scores (lss_norm_), we combine them into a single similarity score (sim) with a parameter *α *to represent the weightage of gene context similarity. Formally, we define the combined similarity score, sim , as follows:

$$\text{sim}\left(g,h\right)=\left(1-\alpha \right)\times \text{s}{\text{w}}_{\text{norm}}\left(g,h\right)+\alpha \times \text{ls}{\text{s}}_{\text{norm}}\left(g,h\right)$$

Using the combined score, we compute the set of bidirectional best hits by sorting all gene pairs in decreasing score and scanning this list once. A gene pair (*g*, *h*) is a bidirectional best hit if sim(*g*, *h*) is strictly greater than sim(*g*, *h*') for all other genes *h*' and sim(*g*, *h*) is strictly greater than sim(*g*', *h*) for all other genes *g*'. This guarantees that the set of bidirectional best hits is always one-to-one.

### Reducing the number of false positives

A drawback of the bidirectional best hit criteria is that it does not take into account the actual similarity between two genes. This may lead to false positives when two genes with very low similarity form bidirectional best hits simply because there are no other similar genes. We found that we can quantify the strength of a particular bidirectional best hit by comparing the similarity of the best hit and the second best hit. Based on this observation, we define the strength of a bidirectional best bit pair (*g*, *h*) as:

$$\text{strength}\left(g,h\right)=\sqrt{\left(\text{sim}\left(g,h\right)-\text{sim}\left(g,h^{\prime }\right)\right)\times \left(\text{sim}\left(g,h\right)-\text{sim}\left(g^{\prime },h\right)\right)}$$

where *h*' is the second best hit of *g *and *g*' is the second best hit of *h*. When there is only one hit, the similarity between a gene and its second best hit is defined to be 0.

We can reduce the number of false positives by only keeping those bidirectional best hits whose strength is greater than a minimum strength threshold *β*.

## Results and discussion

We evaluate our BBH-LS method by applying it to the human, mouse, and rat genomes. For each pair of genome, we compared the performance of BBH-LS, BBH using only normalized Smith-Waterman score (BBH), MSOAR2 [11], InParanoid 4.0 [17], OMA [21], Ensembl Compara [22], and OrthoMCL [14].

By definition, positional homologs are the direct descendants of a single ancestral gene in the most recent common ancestor. It is impossible to confirm the ancestry of a gene unless we have been able to observe its evolution. In practice, we verify our predictions against manually curated gene symbols. This is also the approach used by MSOAR2 [11]. Gene symbols are manually curated based on gene function [23] and they are used by researchers to represent the same gene across different species. The assignment of a gene symbol to a gene is approved by a nomenclature committee to ensure scientific accuracy. However, in the absence of experimentally verified function, genes may be assigned an anonymous and temporary gene symbol based on their sequence/structural similarity to other genes. For the purpose of our experiments, we consider such genes to have not been assigned a meaningful gene symbol.

Using this approach, we can classify the predicted positional homolog pairs into the following three categories:

• true positive: both genes share a common gene symbol

• false positive: gene symbols are completely different

• unknown: either one of the two genes have not been assigned a meaningful(We filter away symbols matching the regular expression "orf" in human genes, "Rik$" or "ˆGM[0-9]+$" in mouse genes, and "ˆLOC[0-9]+$" or "ˆRGD[0-9]+$" in rat genes.) gene symbol

The peptide sequences and locations of genes in each of three genomes were download from the Ensembl Release 60(retrieved from ftp://ftp.ensembl.org/pub/release-60/fasta/ in Nov 2010). There are 20801, 22842, and 22925 genes in the human, mouse and rat genome. We downloaded official gene symbols from the following species specific databases: HUGO Gene Nomenclature Committee(retrieved from http://www.genenames.org in Dec 2010), Mouse Genome Informatics(retrieved from http://www.informatics.jax.org in Dec 2010), and Rat Genome Database (retrieved from http://rgd.mcw.edu in Dec 2010).

### Parameter tuning for BBH-LS

Our scoring scheme uses the parameter *α *to controls the weightage of gene context similarity score. If *α *is 1, then we only use gene context similarity. If *α *is 0, then we only use sequence similarity.

**Figure 4:**
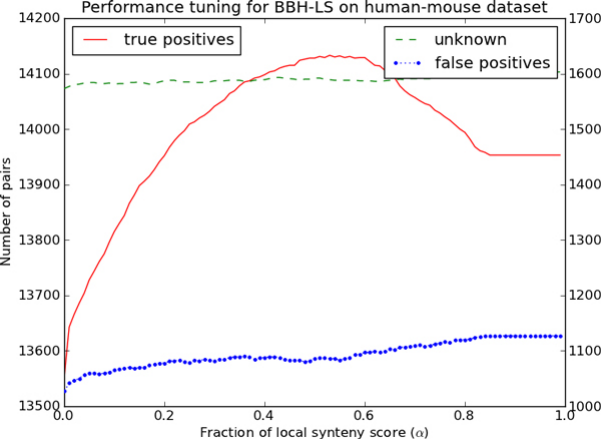
**Determining the optimal combination of sequence and gene context similarity for BBH-LS on the human-mouse dataset**. Performance of BBH-LS for different weightage of gene context similarity to sequence similarity on the *human-mouse *dataset. Left axis indicates the number of pairs of true positives and the right axis indicate the number of unknown pairs and false positives.

**Figure 5:**
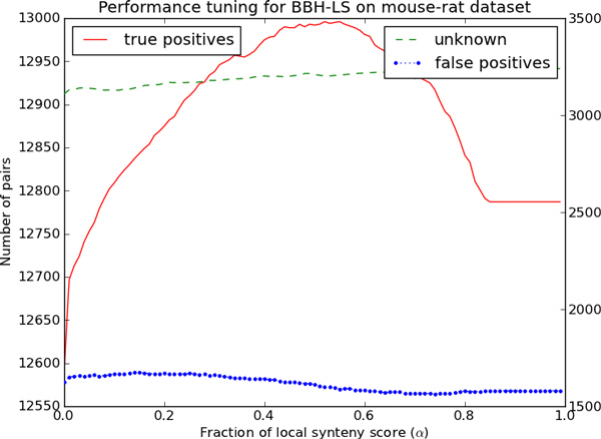
**Determining the optimal combination of sequence and gene context similarity for BBH-LS on the mouse-rat dataset**. Performance of BBH-LS for different weightage of gene context similarity to sequence similarity on the *mouse-rat *dataset. Left axis indicates the number of pairs of true positives and the right axis indicate the number of unknown pairs and false positives.

We want to determine the optimal value of the parameter *α *on the human-mouse and mouse-rat dataset. To do this, we ran BBH-LS on the human-mouse and mouse-rat dataset over a range of values of *α *and tabulated the number of true positives, false positives and unknown pairs for each value. Figure 4 and Figure 5 shows how the number of true positives, false positives and unknown pairs varies as a function of *α *for each dataset.

For the human-mouse dataset (Figure 4) we observe that the number of true positives increases rapidly as *α *increases and then decreases at the same rate after reaching a maximum of 14133 when *α *is 0.53.

However, the number of false positives and unknown pairs also increased slightly as *α *increases. The same general trend for the true positives is observed for the mouse-rat dataset shown in Figure 5 (maximum of 12996 when *α *is 0.52).

We initially thought that the weightage of gene context similarity score should be much lower that of the sequence similarity as many existing methods make use of sequence similarity but not gene context similarity. To our surprise, we found that setting *α *close to 0.50 maximizes the number of true positives for both datasets. In the following experiments, we set *α *as 0.50.

It is estimated that the last common ancestor of human and mouse existed 87 million years ago while the mouse-rat ancestor existed 16 million years ago [24], furthermore there are 297 large scale rearrangement events between human and mouse but only 106 rearrangement events between mouse and rat [25]. Despite the difference in the genomic distance in these two datasets, the best value of *α *is consistently around 0.50. Additional experiments using genomes of varying evolutionary distances will be necessary to determine whether this observation holds more generally.

**Figure 6:**
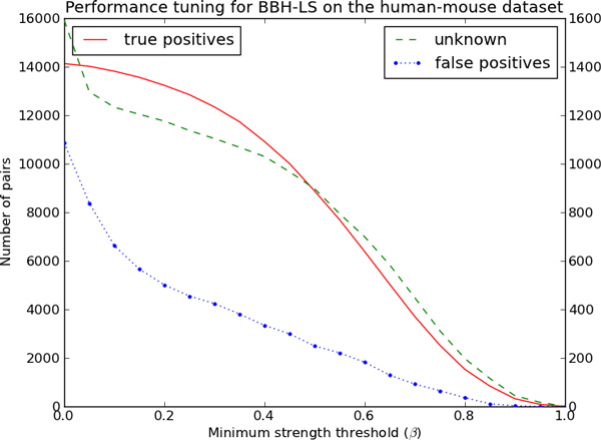
**Reducing the number of false positives predicted by BBH-LS**. Performance of BBH-LS for different strength threshold *β *on the *human-mouse *dataset. Left axis indicates the number of pairs of true positives and the right axis indicate the number of unknown pairs and false positives.

Similarly, we considered the effect of the strength threshold *β *using the human-mouse dataset. Figure 6 shows how the number of true positives, false positives and unknown pairs varies as a function of *β*. All three quantities decrease to zero as the threshold increases. Importantly, the number of true positives decreases slowly for small values of *β *while the number of false positives and unknown pairs drops significantly. This shows that our definition for the strength of a BBH pair is effective at reducing the number of false positives without too much effect on the number of true positives.

### Performance on human-mouse-rat dataset

We obtained the output of the methods in our comparison by running the respective programs on the input data, except for OMA and Ensembl Compara as we did not have access to the programs. We downloaded the orthologs predicted by OMA (retrieved from http://omabrowser.org in Dec 2010) and Ensembl Compara(retrieved from http://www.ensembl.org in Dec 2010) from their respective websites.

#### Analysis of one-to-one pairs

In this analysis, we only focus on one-to-one pairs. InParanoid, OMA, and Ensembl Compara produces pairs of orthologous groups instead of one-to-one positional homolog pairs. We get ortholog pairs by post-processing the output. InParanoid builds its groups from pairs of seed orthologs, we extract the seed orthologs from each group. For OMA, Ensembl Compara, and OrthoMCL, we use only the one-to-one groups.

**Figure 7:**
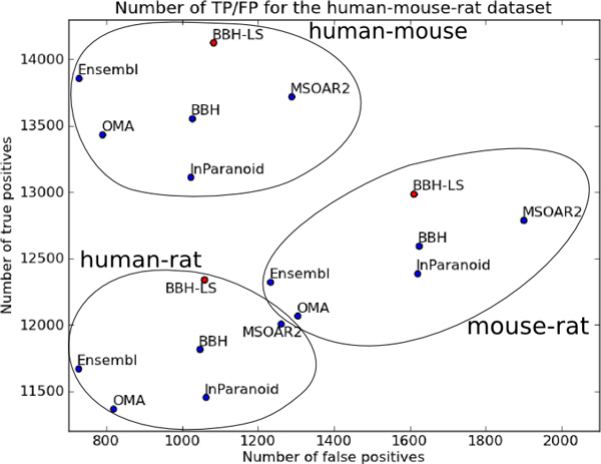
**Comparing the number of true positives and false positives for six different methods on three datasets using only one-to-one pairs**. Plot of number of true positives vs number of false positives in the output of BBH-LS (*α *= 0.50, *β *= 0.00), BBH, MSOAR2, InParanoid, OMA, and Ensembl Compara for the human-mouse, human-rat, and mouse-rat dataset. Only the one-to-one pairs were used in this analysis. For InParanoid, each many-to-many pair is converted to a one-to-one pair by extracting the seed ortholog pair. For OMA and Ensembl, the many-to-many pairs are discarded.

Figure 7 shows the number of true positives and false positives for each method on three datasets. The results for OrthoMCL were not included in Figure 7 as is an outlier; for human-mouse dataset OrthoMCL has 8936 true positives and 498 false positives, for human-rat dataset there are 7409 true positives and 530 false positives, and for mouse-rat dataset there are 7812 true positives and 819 false positives.

For the human-mouse dataset, BBH-LS (*α *= 0.50, *β *= 0.00) identified the largest number of true positives (14128), followed by Ensembl Compara (13856), and MSOAR2 (13718). InParanoid which uses BLAST to compute sequence similarity does significantly worst that BBH using normalized Smith-Waterman alignment scores. In terms of the number of false positives, the methods we evaluated fall into three categories: low false positives (OrthoMCL, OMA, Ensembl Compara), medium false positives (InParanoid, BBH, BBH-LS), and high false positives (MSOAR2). We can reduce the number of false positives to 838 (low false positives), by increasing *β *to 0.05. The corresponding number of true positives is 14018, which is still the highest among all the methods compared. OMA and Ensembl Compara performed surprisingly well given that we only consider the one-to-one groups that were generated.

The results for the human-rat dataset shown in Figure 7 is similar to that of the human-mouse data except that the number of true positives produced by Ensembl Compara and OMA has decrease relative to the other methods, but Ensembl Compara still has more true positives than InParanoid. For the mouse-rat dataset (Figure 7), OMA and Ensembl Compara is now worse than InParanoid. Another interesting characteristic of the mouse-rat dataset is the higher number of false positives, roughly doubled that of the human-mouse or human-rat dataset for all the methods.

Overall, in all three experiments, our BBH-LS method consistently produced the highest number of true positives as validated using gene symbols with a medium level of false positives. The number of false positives can be further reduced by removing BBH pairs with low strength.

**Figure 8:**
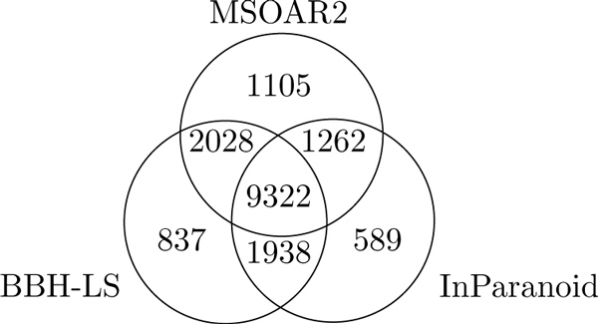
**Overlap between the true positives of BBH-LS, MSOAR2, and InParanoid**. Venn diagram showing the overlap between the true positive one-to-one pairs reported by BBH-LS, MSOAR2, and InParanoid for the human-mouse dataset.

Figure 8 shows a more detailed comparison of the true positives reported by BBH-LS, MSOAR2, and InParanoid. A total of 17081 true positives pairs are identified by at least one of the three methods and 57.5 percent (9322/17081) are identified by all three methods. Therefore, only slightly over half of the true positive pairs exhibit strong signals and are easy to detect. The rest require the combination of a number of different sources of information.

#### Analysis of one-to-one and many-to-many pairs

The previous analysis is biased against methods that produce many-to-many pairs as we excluded all non one-to-one pairs of orthologous groups. In this section, we consider a different definition of true positives and false positives that takes into account many-to-many pairs.

Using the official gene symbol of each gene, we classify each many-to-many pair into the following three categories:

• true positive: there exist a pair of genes (one from each group) that share a common gene symbol

• false positive: all genes have been assigned gene symbols, but there is no pair of genes with a common symbol

• unknown: neither a true positive nor a false positive

**Figure 9:**
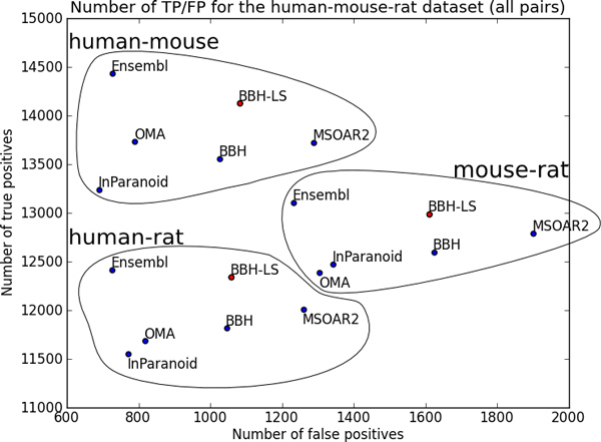
**Comparing the number of true positives and false positives for six different methods on three datasets using all pairs**. Plot of number of true positives vs number of false positives in the output of BBH-LS (*α *= 0.50, *β *= 0.00), BBH, MSOAR2, InParanoid, OMA, and Ensembl Compara for the human-mouse, human-rat, and mouse-rat dataset.

We plot the number of true positives versus the number of false positives for all six methods (excluding OrthoMCL as it is an outlier) on all three datasets and obtained Figure 9. Note that the position of BBH-LS, BBH, and MSOAR2 is the same as in Figure 7 since these methods only predict one-to-one pairs. Only the position of InParanoid, OMA, and Ensembl Compara changed as these methods were designed to predict many-to-many orthologous groups.

InParanoid has much fewer false positives and about the same number of true positives as in the previous analysis. Most of the false positives are now classified as unknown, as a many-to-many pair can only be a false positive if all of the genes have been assigned gene symbols. In contrast, the number of false positives for OMA and Ensembl Compara did not change significantly, but the number of true positives have gone up.

In particular, we note that in this analysis, Ensembl Compara has the highest number of true positives, with BBH-LS coming in at a close second. This is expected as this method of analysis is biased towards methods that generates many-to-many pairs. Recall that only one out of the *n *× *m *possible gene pairs in a *n*-to-*m *pair need to have a common gene symbol for the *n*-to-*m *pair to be considered a true positive.

#### Two cases where gene context similarity made a difference

**Figure 10:**
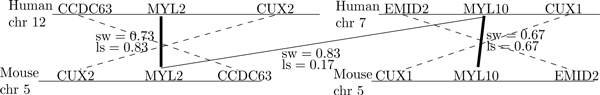
**Incorrect pairing due to similar sequence similarity corrected by BBH-LS**. BBH erroneously paired MYL2 (human) to MYL10 (mouse) because of high Smith-Waterman score, this was corrected by BBH-LS with the help of local synteny score. Bold edges are the pairing from BBH-LS, thin edges are the pairing from BBH, sw = normalized Smith-Waterman score, lc = normalized local synteny score.

**Figure 11:**
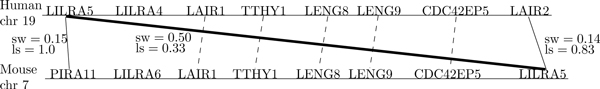
**BBH-LS mislead by high local synteny**. BBH-LS paired LILRA5 (human) with PIRA5 (mouse) and LAIR2 (human) with LIRA5 (mouse) due to the high local synteny produced by the five pairs of genes in between. The correct pairing should be LILRA5 (human) with LILRA5 (mouse) and this was picked up by BBH using just the normalized Smith-Waterman score.

In the following, we illustrate a number of specific instances where gene context similarity made a significant differences. Figure 10 shows an instance where a large difference in the gene context similarity helped to identify the positional homolog among genes with similar sequence similarity. There are a total of 48 cases where gene context similarity helped to converted a false positive found by BBH to a true positive found by BBH-LS. However, in four cases, the local synteny score caused a true positives identified by BBH to become a pair of false positives. Figure 11 illustrates one of these cases.

## Conclusion

The ORTHOLOG ASSIGNMENT problem is challenging in practice due to gene duplications and gene loss. Several sophisticated methods, which make use of complex heuristics (InParanoid) or require solving computationally hard problems (MSOAR2), have been proposed to tackle this problem. However, we show in this paper that the simple bidirectional best hit heuristic, coupled with a scoring scheme that combines both sequence and gene context similarity, is surprisingly good at identifying positional homologs. In all three pairwise comparison between human, mouse, and rat genomes, our BBH-LS method identified the most number of positional homolog (as validated using gene symbols) with a medium number of false positives.

We are current investigating the application of our method for ortholog assignment in plant genomes.

## Competing interests

The authors declare that they have no competing interests.

## Authors' contributions

MZ developed the BBH-LS algorithm, carried out the experiments, and drafted the manuscript. HWL contributed to research discussions and revised the manuscript. All authors read and approved the final manuscript.
